# Describing a developing hybrid zone between red wolves and coyotes in eastern North Carolina, USA

**DOI:** 10.1111/eva.12388

**Published:** 2016-06-01

**Authors:** Justin H. Bohling, Justin Dellinger, Justin M. McVey, David T. Cobb, Christopher E. Moorman, Lisette P. Waits

**Affiliations:** ^1^ Department of Fish and Wildlife ResourcesUniversity of IdahoMoscowIDUSA; ^2^ School of Environmental and Forest SciencesUniversity of WashingtonSeattleWAUSA; ^3^ Department of Forestry and Environmental ResourcesNorth Carolina State UniversityRaleighNCUSA; ^4^ North Carolina Wildlife Resources CommissionRaleighNCUSA

**Keywords:** conservation‐reliant species, endangered species, genetic cline, genetic introgression, noninvasive genetic sampling

## Abstract

When hybridizing species come into contact, understanding the processes that regulate their interactions can help predict the future outcome of the system. This is especially relevant in conservation situations where human activities can influence hybridization dynamics. We investigated a developing hybrid zone between red wolves and coyotes in North Carolina, USA to elucidate patterns of hybridization in a system heavily managed for preservation of the red wolf genome. Using noninvasive genetic sampling of scat, we surveyed a 2880 km^2^ region adjacent to the Red Wolf Experimental Population Area (RWEPA). We combined microsatellite genotypes collected from this survey with those from companion studies conducted both within and outside the RWEPA to describe the gradient of red wolf ancestry. A total of 311 individuals were genotyped at 17 loci and red wolf ancestry decreased along an east–west gradient across the RWEPA. No red wolves were found outside the RWEPA, yet half of individuals found within this area were coyotes. Hybrids composed only 4% of individuals within this landscape despite co‐occurrence of the two species throughout the RWEPA. The low proportion of hybrids suggests that a combination of active management and natural isolating mechanisms may be limiting intermixing within this hybrid system.

## Introduction

Fluctuations in environmental conditions can alter patterns of contact between reproductively compatible species. As ranges shift and individuals interact potential outcomes can range from genetic homogenization to reproductive isolation (Jiggins and Mallet 2000; Crispo et al. 2011; Robbins et al. 2014). Predicting the consequences of such contact has developed into an important conservation issue. Human alterations to the environment can increase the rate at which previously isolated species come into contact and hybridize (Rhymer and Simberloff 1996; Seehausen et al. 2008; Crispo et al. 2011). Hybridization may occur more rapidly than mitigation can be implemented, facilitating genetic swamping, and genomic extinction (Allendorf et al. 2001).

While some human activities may promote hybridization, active management for conservation purposes could limit the degree of interbreeding and genetic introgression. There are few examples where wild populations are actively manipulated to manage against the threats posed by interspecific hybridization, and even fewer where this management has successfully limited introgression. The interaction between natural processes, anthropogenic disturbance, and conservation management is poorly understood but potentially drive the fate of emerging hybrid systems.

**Figure 1:**
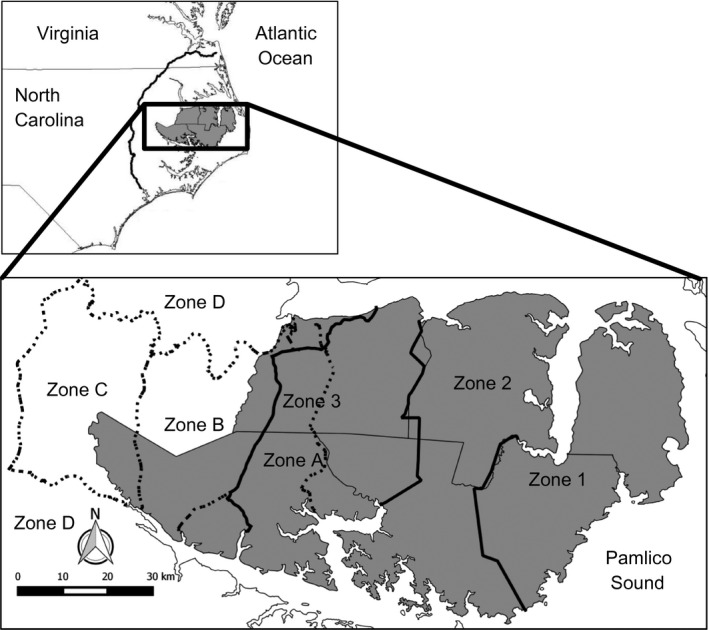
Map of the Red Wolf Experimental Population Area (RWEPA) and the associated study design. The areas shaded in gray represent the five counties that compose the RWEPA, and the solid black lines are the boundaries of the three management zones. The dashed lines indicate the boundaries of the sampling zones designated for the 2010 scat survey. Note that the western boundaries of Zone 3 and Zone A overlap for most of their lengths. The inset is a map of eastern North Carolina and the RWEPA. The solid black line in the inset indicates the western boundary of the 2008 scat survey (Zone D).

The critically endangered red wolf (*Canis rufus*) provides an opportunity to examine the development of a hybrid zone between two expanding populations actively managed for conservation purposes. Red wolves were historically found across what is now the eastern United States (Nowak 2002). Range contraction due to human persecution and habitat loss facilitated widespread hybridization with coyotes (*C. latrans*), causing the species to nearly disappear into a hybrid swarm (McCarley 1962; Paradiso and Nowak 1972; Parker 1988). In the 1970s, the US Fish and Wildlife Service (USFWS) captured all remaining wild red wolves to initiate a captive breeding program. In 1987, red wolves were reintroduced into the Albemarle Peninsula (Fig. 1) in eastern North Carolina (USFWS 1986; Phillips and Parker 1988). Over the past decade, the population has numbered around 60–100 individuals (Bartel and Rabon 2013; Gese et al. 2015).

Following the extirpation of the red wolf, the coyote expanded its range throughout the eastern United States. In 1993, the first hybridization event between a reintroduced red wolf and coyote occurred (Kelly et al. 1999; Adams 2006). In response, the USFWS in partnership with the Red Wolf Recovery Implementation Team (RWRIT) formed an adaptive management plan to limit hybridization (Stoskopf et al. 2005). Management practices included euthanizing or sterilizing coyotes and hybrids, removing hybrid litters from the landscape, and encouraging breeding opportunities between red wolves (Gese et al. 2015). The Albemarle Peninsula has been designated as the Red Wolf Non‐Essential Experimental Population Area (RWEPA) and divided into three distinct management zones: Zone 1, 2, and 3. The intensity of management across these zones varies and creates a core red wolf population in the eastern portion of the Peninsula (Zone 1). Management is less‐intense further west closer to the mainland (Zone 3) due to limited resources, creating a gradient of management from east to west. This program has been successful in limiting hybridization and preventing genetic swamping. Noninvasive genetic surveys and active trapping within the RWEPA suggest a dominance of red wolf genotypes with isolated instances of hybridization (Adams et al. 2003, 2007; Gese et al. 2015). Tracking reproductive events through a reconstructed pedigree has revealed only one hybridization event, the initial litter from 1993, resulted in the introgression of coyote DNA into the red wolf population (Adams 2006; Bohling et al. 2013). There have been substantially more documented red wolf litters than hybrid litters (Bohling and Waits 2015; Gese et al. 2015).

Beyond the western edge of the RWEPA, however, there has been limited investigation as to whether dispersing red wolves are intermixing with coyotes and creating a genetic cline across this region. Bohling and Waits (2011) conducted a noninvasive genetic survey (NIS) of scats across a 22 000 km^2^ region surrounding the RWEPA and found no red wolves and little evidence of red wolf introgression into the coyote population. However, this survey was designed to provide a coarse assessment of canid ancestry and covered ≈1% of potential sampling locations. It did not include the RWEPA or much of the area immediately surrounding it, limiting inference into spatial genetic turnover.

Our goal in this study was to examine the cline of red wolf genetic ancestry radiating from the core of the RWEPA into the surrounding mainland and assess the distribution of red wolves, coyotes, and their hybrids across the landscape. We hypothesized that this red wolf–coyote system would exhibit a trimodal hybrid zone distribution (Jiggins and Mallet 2000). We expected limited introgression of coyote genetic material into the core red wolf area. Conversely, beyond the RWEPA, we anticipated limited red wolf introgression as genetic inputs from red wolves would likely be swamped by the larger coyote population. At the edge of the RWEPA where management is less rigorous, we expected hybridization to be prevalent and hybrids more populous than either parental species. To test this hypothesis, we used NIS to assess the gradient of red wolf ancestry across the landscape. By describing the composition of the hybrid zone, we can understand patterns of hybridization during the early stages of contact between two canid species subjected to both positive and negative human manipulation.

## Methods

### Study area and sampling methods

The study area was selected to overlap the potential zone of contact between the managed red wolf population and the mainland coyote population. With little evidence of red wolf introgression outside the RWEPA (Bohling and Waits 2011), we restricted the size of our study area to intensify sampling around the potential contact zone. We selected portions of five counties (Washington, Beaufort, Martin, Edgecombe, and Pitt) in north‐eastern North Carolina that compose or are adjacent to the RWEPA (Fig. 1). This area was bordered by the Roanoke and Tar Rivers on the north and south, respectively, on the east by NC Route 99, and on the west by US Route 13.

We divided the study area into three zones (Zones A, B, and C) that reflected different intensities of USFWS management and red wolf colonization. The eastern most portion of the study area was designated as Zone A and contained several known red wolves and coyotes at the time of the survey. A majority of this zone overlaps the red wolf RWEPA and constitutes the western limit of red wolf management. Zone B was adjacent to the boundary of the RWEPA but is not intensively monitored by the USFWS. Red wolves and coyotes originally captured within the RWEPA have been known to disperse into this area. Zone C was the furthest west of the zones, has never been monitored by the USFWS, and did not contain any known canids from the RWEPA. Portions of Zones B and C were included in the survey conducted by Bohling and Waits (2011). Major north–south running highways served as boundaries between these three zones.

We used NIS of fecal material (scat) to collect genotypes from canids in this area. Sampling occurred from January through March 2010, which corresponds to the breeding season of red wolves. We collected scats along rural nonpaved roads that traversed farm fields, managed forests, and protected areas. We attempted to survey a similar number of kilometers of roads within each zone. Unpaved roads were identified using maps, US Census Bureau data (U.S. Census Bureau 2000), and information provided by USFWS biologists. A small piece of fecal material (~1 cm^2^) from the outside surface of the scat was removed from each scat using metal tweezers and then placed in a 2.0‐mL screw‐top tube containing 1.2 mL DET buffer (Frantzen et al. 1998). Tweezers were exposed to an open flame before and after use to prevent cross‐contamination between samples.

### Molecular methods

Scat samples were transported to the Laboratory for Ecological, Evolutionary, and Conservation Genetics at the University of Idaho and extracted in a laboratory dedicated to low‐quality DNA samples using the QiAmp Stool Kit (Qiagen, Valencia, CA, USA). We screened samples for species identification using a PCR‐based mitochondrial DNA control region fragment analysis test (Onorato et al. 2006). This test can differentiate between scats deposited by coyotes and red wolves from those deposited by dogs and gray wolves. It cannot differentiate between coyote and red wolf scats because these species produce similarly sized fragments. For samples that tested positive for *Canis* mitochondrial DNA, we screened them at 17 polymorphic nuclear microsatellite loci following the methods of Bohling and Waits (2011). Each sample was initially screened in a nine loci multiplex, and samples that amplified at five or more loci were re‐amplified to verify observed alleles. Heterozygous genotypes were only accepted if each allele was observed in two independent PCRs and homozygous genotypes if they was observed in three independent PCRs. Samples were only included in further analyses if they were genotyped at six or more loci. The probability of identity for siblings (PID_Sibs_) at six loci was sufficiently low (0.003–0.006) in the red wolf population to differentiate individuals.

Once scats were genotyped at six or more loci, we ran a matching analysis using GenAlEx (Peakall and Smouse 2012) to determine whether those genotypes matched any known red wolves or coyotes from the RWEPA. We also compared those scat genotypes to individuals identified during the previous survey in 2008 and additional scat surveys conducted within the RWEPA (see below). Scats with genotypes that matched known canids were assigned to that individual; all other scats were amplified at an additional set of eight microsatellite loci to provide additional resolution (Bohling and Waits 2011). We also identified matches among the scat genotypes. Error rates for allelic dropout and false alleles were similar to those we observed previously (Bohling and Waits 2011), and our replication requirements were designed to detect and minimize genotyping errors. No locus warranted exclusion from analysis based on error rates.

### Measures of HWP and genetic diversity

To evaluate the genetic composition of canids across this landscape, we pooled together genetic samples from multiple sources to provide greater coverage of the region. Within the RWEPA, two independent studies were conducted examining the dietary habits of red wolves and coyotes using scat samples (Dellinger et al. 2011; McVey et al. 2013). The genetic methods used for those surveys were identical to those of this study. Combined these two studies covered the entire range of the RWEPA and extended into portions of our designated study area. Also, we included individuals genotyped by Bohling and Waits (2011). Incorporating all of these samples provided an opportunity to extend the coverage of analysis across the entire gradient of red wolf ancestry, from the core of the red wolf population into the mainland coyote population. From an east–west direction, the zones were as follows: Zone 1, Zone 2, Zone 3, Zone B, Zone C, and Zone D (Fig. 1). We combined Zones 3 and A because of substantial overlap. In later analyses, we state whether an analysis included only the data gathered specifically for this study (2010 scat survey, covering Zones A‐C), the 2008 data from Bohling and Waits (2011) collected outside the RWEPA (hereafter referred to as Zone D), the data from the two dietary studies (collected in RWEPA management Zones 1–3), or a combination of them.

Since the 2010, 2008, and dietary scat surveys featured the same loci, we initially conducted a global test for deviations from Hardy–Weinberg Proportions (HWP) for each of the 17 loci by combining genotypes across all four studies and all sampling zones. The global analysis suggested significant departures from HWP (see Results). We then conducted tests for HWP for each locus after grouping individuals within the RWEPA (Zones 1–3) or outside (Zones B‐D). All tests were conducted using exact tests with 1000 Monte Carlo replicates implemented with the *pegas* package (Paradis 2010) in R 3.2 (R Core Team 2015).

Deviations from HWP can be caused by a variety of processes, ranging from real biological phenomenon to random statistical error. Due to the large number of deviations we observed, we sought to elucidate phenomena that could generate those patterns. We estimated *F*_IS_ and *F*_ST_ for each locus within and outside the RWEPA using the R package *diveRsity* (Keenan et al. 2013). For both the RWEPA (Zones 1–3) and outside region (Zones B‐D), we performed a linear regression of *F*_IS_ and *F*_ST_ values for each locus to evaluate the potential for a Wahlund effect (Waples 2015). The presence of two distinct genetic groups (e.g., red wolf and coyote) can lead to the correlation between *F*_IS_ and *F*_ST_ across loci.

We calculated observed (*H*_O_) and unbiased expected heterozygosity (*H*_E_) and *F*_IS_ per sampling zone basis. Unbiased 95% confidence intervals for *F*_IS_ were generated using 1000 bootstrap replicates. Similarly, we estimated zone‐specific measures of allelic richness (A_R_) using 1000 resamples with replacement. All these metrics were estimated using *diveRsity*.

### Evaluating the genetic gradient

Individuals were classified into zones based upon the location of scats they deposited. Some individuals were detected multiple times (see Results): for these individuals, we calculated the mean X–Y coordinate based on all observations using QGIS (QGIS Development Team 2015). For all genotypes detected via NIS, we used the Bayesian clustering program STRUCTURE (Pritchard et al. 2000; Falush et al. 2003) to estimate ancestry. We implemented the admixture model with correlated allele frequencies and no prior population information. The burn‐in period was 100 000 reps followed by 1 000 000 MCMC reps. *K* ranged from 1 to 10 and we used the approach of Evanno et al. (2005) to determine the most likely value of *K*. Composite *q*‐values from five iterations of each *K*‐value were generated using CLUMPP (Jakobsson and Rosenberg 2007). We did not include reference genotypes of red wolves and coyotes in this analysis. Because samples were obtained noninvasively, we had no *a priori* means to determine which generated clusters corresponded with the red wolf and coyote population. However, a number of genotypes obtained from scats matched known wolves, coyotes, and hybrids. Thus, we could determine which cluster corresponded with each known group. We calculated the mean red wolf ancestry for each zone using the *q*‐values produced by STRUCTURE. We performed an anova to test the hypothesis of equal red wolf ancestry across all six zones coupled with Fisher's LSD test to group zones based on similarity of means.

To classify individuals to parental groups, we applied a *q*‐value cutoff of 0.875 to denote a red wolf and less than 0.125 to denote a coyote. Values in‐between 0.875 and 0.125 were considered hybrids. These values were selected based on ancestry thresholds developed by the USFWS for managing red wolves (see Stoskopf et al. 2005). In addition, they match theoretical values of ancestry expected after two generations of backcrossing, after which it becomes difficult to distinguish pure parentals from backcrosses using *q*‐values (Bohling et al. 2013). With those thresholds, we were able to estimate the number of red wolves, coyotes, and hybrids detected overall and per zone. Based on the overall proportion of hybrids in the global dataset (Zones 1‐D), we performed a *χ* ^2^‐test comparing the observed distribution of hybrids across the six zones to an expected distribution under equal proportions.

Various combinations of parental groups, hybrids, and backcrossed individuals can generate similar system‐level estimates of genetic ancestry. For example, an equal 50/50 mix of 100% pure individuals from each parental group would produce the same global mean ancestry value (*q *=* *0.50) as a population composed entirely of F1 hybrids. Thus, the distribution of *q*‐values is relevant for assessing the pattern of hybridization. We tested several hypotheses of red wolf–coyote interactions against observed data. These hypotheses were based on modeled predictions of the probability of persistence for the red wolf population under various scenarios of hybridization performed by Fredrickson and Hedrick (2006). They simulated persistence based on the various levels of intermixing and mate choice: comparing their predictions to our empirical data provides insight into processes that may govern hybridization. Such a comparison also assesses whether hybridization is occurring at a rate that threatens the viability of the red wolf population.

The first scenario assumed random breeding between coyotes and red wolves; the second incorporated weak positive assortative mating; and the third included both assortative mating and territorial challenges by red wolves against coyotes. With these three scenarios they predicted the proportion of hybrids in the population at various future time points (e.g., 10, 20, 30, … 100 years). Because they began their simulation analyses at an initial contact point between red wolf and coyote populations, we compared our data to their predictions after 20 years of interaction given that the reintroduction program began in 1987 and we collected data from 2008–2010. In the random mating scenario, they predicted that after 20 years 38.6% of the canid population would be composed of hybrids. For the assortative mating scenario, they predicted 27.6% would be hybrids; red wolf challenges, 18.9%.

Based on the results of STRUCTURE, we were able to estimate a global mean value of red wolf ancestry across all six zones (26.3%, see Results). We calculated the number of hybrids, coyotes, and red wolves that would have to be present in each scenario to produce that value. In other words, considering the number of hybrids expected in each simulated scenario, we estimated how many red wolves and coyotes would have to be present to produce that same global value of red wolf ancestry. Across all six zones we detected 311 individuals, 180 of which were in Zones 1–3. Results from this study and Bohling and Waits (2011) show a lack of red wolf ancestry beyond the RWEPA. Thus, we expected any significant interaction between the two species to occur primarily within the RWEPA and we adjusted our estimates to reflect the lack of red wolves in periphery areas. For an example, in the random mating scenario, 69 individuals in Zones 1–3 would be expected to be hybrids. The remaining 111 individuals must be some combination of red wolves and coyotes that would produce the mean red wolf ancestry (43.7%) actually observed within the RWEPA. If *T* is the total number of individuals detected, *H* is the number of hybrids, and *R* the number of the red wolves, the calculation becomes 0.437*T =* *0.5*H + *R*. With 69 hybrids, the estimate becomes 0.437*180 = 0.5*69 + *R*, or *R *≈* *44. The number of coyotes would be 180‐69‐44, or ≈67. We performed such estimates for all three scenarios from Fredrickson and Hedrick (2006) plus one involving spatial mixing between pure parental genotypes with no interbreeding (Table 1). For the combined area of Zones B, C, and D, we estimated the number of hybrids that would be present to produce the small amount of observed red wolf ancestry (see Results). This was the same for all scenarios: 131 individuals at 2.4% observed red wolf ancestry translates to six hybrids and 125 coyotes. We then simulated genotypes for the three classes (red wolf, coyote, and hybrid) using HybridLab (Nielsen et al. 2006). Genotypes from known red wolves and coyotes from eastern North Carolina were used to generate the genotypes. For all scenarios, we assumed an equal number of F1, F2, F1xcoyote, and F1xred wolf backcrosses among our hybrid class.

**Table 1:** Number of simulated genotypes for various scenarios of red wolf–coyote hybridization. Each scenario was designed to produce an overall ancestry value that matched the studywide observed red wolf ancestry of 26.3%. The scenarios are based on Fredrickson and Hedrick (2006) after 20 years of secondary contact. Genotypes were simulated using HybridLab

Scenario	Hybrid proportion	Number of simulated genotypes					
		Red wolves	Coyotes	F1	F2	F1xRW backcross	F1xCoy backcross
Random mating	0.386	44	192	19	18	19	19
Assortative mating	0.276	54	202	14	14	14	14
Red wolf challenges	0.189	62	209	10	10	10	10
Spatial mixing^a^	0	82	229	0	0	0	0

a The spatial mixing scenario was not part of Fredrickson and Hedrick's study.

We analyzed these simulated genotypes in STRUCTURE using the same parameters as the initial analysis at *K *=* *2. Composite *q*‐values were again generated using CLUMPP based on ten iterations. We compared the distribution of *q*‐values to the observed data using a Kolmogorov–Smirnov test (Nielsen et al. 2003; Latch et al. 2011). For each distribution of *q*‐values produced by the simulated scenario, we fitted a logistic function to compare the steepness of their respective curves. Using the *nls* function in R, we fitted the model:

$$y=a/(1+e^{-c(x-b)})$$

to the distribution of *q*‐values (*x*) using an iterative procedure. The steepness of the curve (*c*) reflects the abundance of intermediate *q*‐values (i.e., hybrids) and provides perspective to compare our empirical dataset to the simulated scenarios.

## Results

### Sample distribution

In 2010, we collected 500 scats from across the study area over the 2‐month period (Table S1). Samples were not evenly distributed across all three zones: the largest proportion was collected from Zone A (45.6%) and the second most from Zone B (38.4%). Only 15.8% of the scats were collected in Zone C. This unequal distribution was also reflected in the amount of roads we sampled: over 250 km of roads were sampled in both Zones A and B, respectively, whereas only 110 km were sampled in Zone C.

Two hundred sixty‐four scats were identified as *Canis* using the mitochondrial fragment test: 201 possessed a red wolf/coyote fragment and 63 a gray wolf/dog fragment. Of these 264 scats, 156 were amplified at six or more loci with the average number of loci amplified per sample being 9.8 (range 6–17). Regrouping the genotypes revealed 87 individuals among our dataset, only one of which matched a known coyote that had been captured by USFWS biologists. Nine of these genotypes matched individuals that had been identified during scat surveys for the diet analysis projects. The remaining 77 were unique to this study. These individuals were genotyped at an average of 12.5 loci each (range 6–17).

When scats collected for other studies were included in the dataset, a combined total of 2665 scats were obtained across all six zones and a total of 763 were genotyped at six or more loci. Of these 763 scats, 291 produced genotypes that matched a known individual from within the RWEPA. These 291 scats were assigned to 58 known red wolves and six known coyotes. The remaining 472 scat genotypes did not match any known individuals and regrouping these samples revealed a total of 263 unique individual genotypes. Each of these unique individuals was genotyped at an average of 11.3 loci (±3.1 SD) with a range from six to 17.

We were able to obtain locations for 311 individuals that had been genotyped via NIS. The largest number of individuals was detected in Zone 3, which contained nearly four times as many individuals as the zone with the fewest (Zone C). This was also the only zone to be sampled by the 2010 survey and both dietary studies. Nine individuals were detected in multiple zones, of which seven were known red wolves or coyotes with home ranges that overlapped the boundaries of two zones. Two were unknown individuals discovered along the boundary of Zones 3 and B that deposited scats in both.

### HWP and genetic diversity

The global dataset deviated strongly from neutral HWP (*P *<* *0.001). When individuals were divided between those within the RWEPA and those outside, the global tests for both regions deviated from HWP but did so with differing severity. Within the RWEPA, only one locus followed neutral expectations. Locus‐specific values of *F*_IS_ and *F*_ST_ within the RWEPA were positively correlated (*r *=* *0.74, *P *=* *0.007, Fig. S2A), indicative of a Wahlund effect. The intercept of the linear model was 0.04 with a slope of 1.96. In contrast, nine of the 17 loci deviated from HWP outside the RWEPA (*P *<* *0.05). There was no correlation between *F*_IS_ and *F*_ST_ (*r *=* *0.07, *P *=* *0.8, Fig. S2B).

For the global dataset, *H*_O_ was less than *H*_E_ (0.677 vs 0.756), resulting in a positive value of *F*_IS_ (0.092). When samples were grouped by zone, only Zones 1 and 2 produced estimates of heterozygosity less than the global value (Fig. S2A). Zones 1, B, and D had *F*_IS_ less than the global mean and only for Zones 1 and B did the 95% CI around the point estimate not overlap zero (Fig. S2C). A_R_ was lowest in Zone 1, and it was the only zone for which the 95% CI did not overlap the others (Fig. S2B). Zone 3 had the highest value of A_R_.

### Red wolf ancestry gradient

**Figure 2:**
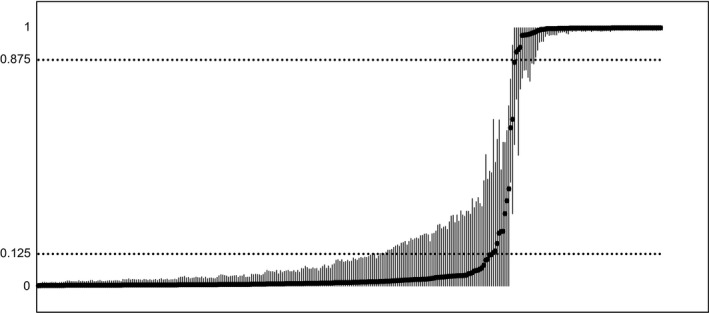
Distribution of *q*‐values produced by STRUCTURE at *K *=* *2 for all individuals detected via noninvasive genetic sampling. Each *q*‐value is surrounded by a 90% credibility interval. The vertical axis denotes the *q*‐value estimated for the red wolf cluster identified by STRUCTURE. Individuals are sorted along the horizontal axis in ascending order according to their *q*‐value.

**Figure 3:**
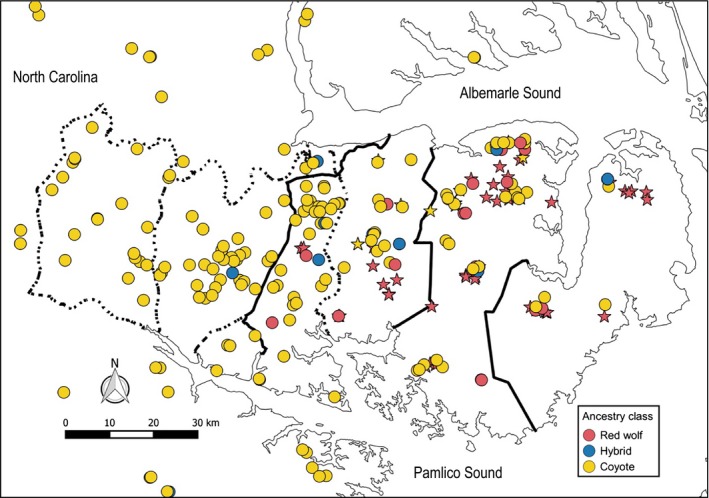
Distribution of individuals detected across the study area and their associated amount of red wolf ancestry. Each point represents a different individual, and each color reflects its classification based on red wolf ancestry. Stars represent individuals that had previously been captured and genotyped; circles denote new individuals identified via NIS. ‘Red wolf’ refers to individuals with a STRUCTURE *q*‐value great than 0.875 for the red wolf cluster; ‘Hybrid’ between 0.125 and 0.875; ‘Coyote’ less than 0.125. The solid black lines are the boundaries of the three management zones, and the dashed lines indicate the boundaries of the sampling zones designated for the 2010 scat survey. Note that this map does not cover the entire extent of Zone D: only individuals that fit within this frame are represented on the map.

Based on the Δ*K* method, most likely number of clusters was two (Fig. S1). One cluster estimated by STRUCTURE contained the genotypes of the 58 known red wolves and the other the six known coyotes. Based on our 0.125 *q*‐value threshold, we identified 75 red wolves, 224 coyotes, and 12 hybrids across all six sampling zones (Fig. 2). Within the RWEPA, there were 96 coyotes and nine hybrids accompanying the 75 red wolves. No red wolves were found west of the RWEPA. Red wolf genotypes were predominant in Zones 1 and 2 but composed a minority of the genotypes in all other zones (Fig. 3). Hybrid genotypes never composed more than 8% of the individuals in any zone and were only more common than red wolf genotypes in Zones B and D. Globally, hybrids composed about 3.86% of individuals and their distribution across the six zones did not differ from that expected under equal proportions (*χ* ^2 ^= 18, *P *=* *0.263).

**Figure 4:**
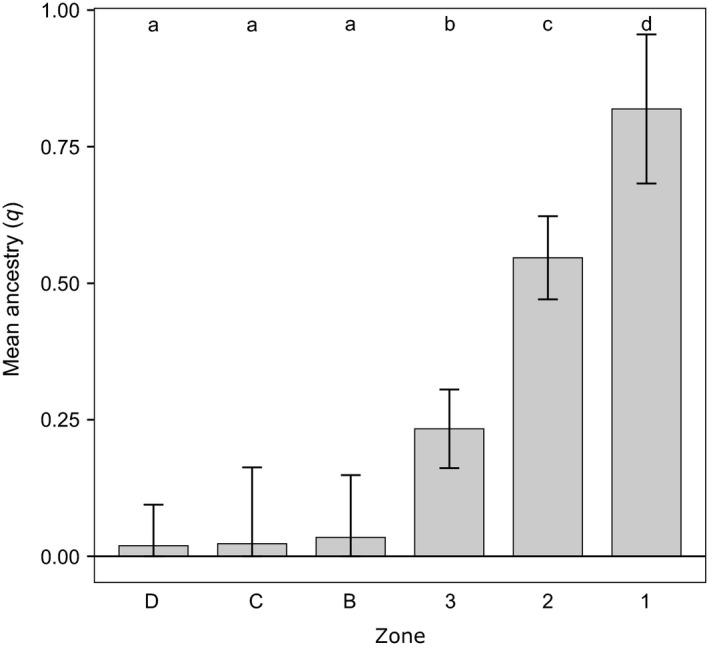
Average level of red wolf ancestry for each geographic zone. These values were determined by averaging the amount of red wolf ancestry across all individuals detected in each zone. Each value is surrounded by its corrected 95% confidence interval. Lower case letters indicate groups of zones that could not differentiated using the Fisher's LSD test. Note that the distribution of the zones on this graph follows geographic distribution across this system with Zone D as the western most zone and Zone 1 as the eastern most. The distance between the zones on the x‐axis does not reflect their actual geographic distance. Note that sampling Zones 3 and A were combined for this analysis due to substantial spatial overlap.

The average red wolf ancestry for each zone declined along an east–west gradient (Fig. 4). Only in Zones 1 and 2 was the mean red wolf ancestry >50%. The overall anova model deviated from the null hypothesis of equal levels of red wolf ancestry across the entire region (*F *=* *37.28, *P *<* *0.001). The Fisher's LSD test revealed the three zones (1–3) within the RWEPA possessed levels of red wolf ancestry dissimilar from all other zones (Fig. 4). In contrast, levels in the three zones located outside the RWEPA (B–D) were indistinguishable. Even when Zone D was removed from the dataset to eliminate bias potentially caused by its large spatial coverage, there was still unequal red wolf ancestry across the remaining five zones (*F *=* *25.02, *P *<* *0.001).

**Figure 5:**
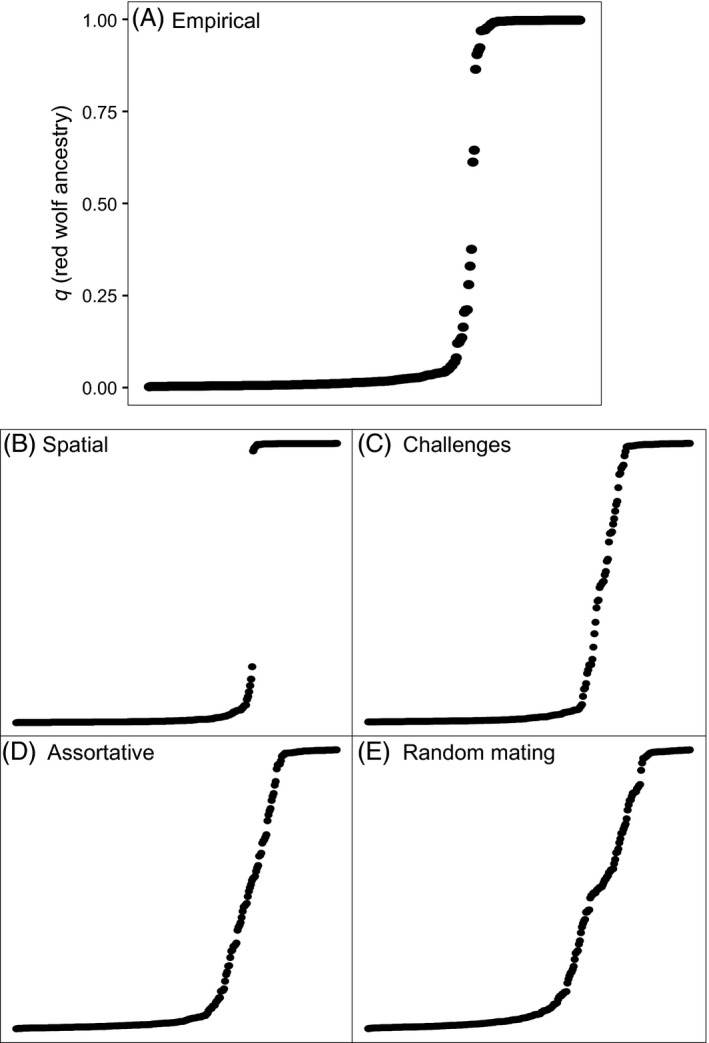
Distribution of *q*‐values representing red wolf ancestry for the empirical data (A) and the four simulated scenarios (B–E). Each point represents an individual, and they are ranked on the horizontal axis in ascending order by their *q*‐value. These ancestry values were produced by STRUCTURE. For the bottom four panels, the vertical axis is unlabeled but it follows the same scale as Panel A.

Kolmogorov–Smirnov tests showed that the distribution of *q*‐values produced under the four simulated scenarios deviated strongly from the empirical dataset (Fig. 5). The spatial mixing, random mating, and assortative mating scenarios produced test statistics (*D*) of 0.225, 0.408, and 0.289, respectively, and *P*‐values less than 3 × 10^−7^. Although also highly different, the challenges scenario was the most similar to the empirical dataset with a test statistic of 0.138 and *P *=* *0.005. As all the analyses were based on the same range of values (e.g., 0 < *q *<* *1), the parameter estimates for the asymptotes (range 0.997–1.052) and midpoints (range 226.529–232.917) of our fitted logistic functions were similar (Table S2). The primary difference was in the slopes. The steepest slope, as expected, was produced by the spatial mixing scenario (*c *=* *4.599). The next steepest was produced by the empirical dataset (*c *=* *0.364) followed by the red wolf challenges scenario (*c *=* *0.109).

## Discussion

### Distribution of hybrids and parental types

Based on our results, hybridization between red wolves and coyotes is infrequent relative to the proportion of the parental groups in the landscape. Such findings run counter to our hypothesis that high levels of introgression would form a trimodal distribution of genotypes, with the greatest abundance of hybrids in the contact zone. Instead, we observed a bimodal distribution with limited numbers of hybrids. Hybrids were evenly distributed across the six zones; we predicted greater abundance in the contact zone, which suggests hybridization may be an isolated local phenomenon rather than a product of the proportion of parental individuals in a given area.

Two separate surveys (2008 and 2010) failed to identify any red wolves and few hybrids outside of the RWEPA. Between 2000 and 2011, there was high annual production of pups (~40–50)(US Fish and Wildlife Service 2007; Gese et al. 2015) and many of these pups were subsequently not captured as adults within the RWEPA. Modeling of population trends and habitat selection reveal a lack of potential space for dispersing wolves within the RWEPA (Sparkman et al. 2011; Dellinger et al. 2013). These conditions should promote red wolf movement and colonization westward, so the lack of red wolf colonization outside of the RWEPA is puzzling.

It is possible that wolves exiting the RWEPA encounter a vast heterogeneous landscape where all suitable blocks of habitat are occupied by coyotes. Roth et al. (2008) simulated spatial interactions between red wolves and coyotes and suggested the presence of coyotes could limit red wolf colonization by occupying potential territory. In a comparable hybrid system, Benson and Patterson (2013) observed high territoriality and low spatial overlap between packs of eastern wolves (*C. lycaon*), coyotes, and gray wolves in south‐western Ontario. If such a relationship holds true in North Carolina, spatial segregation between canid species may limit red wolf colonization. However, field observations suggest red wolves frequently displace sterile placeholder coyotes and hybrids (Gese and Terletzky 2015). These contrasting scenarios suggest more research is needed to understand whether competitive interactions among hybridizing canid species has limited red wolf colonization.

Another factor likely limiting the ability for dispersing wolves to establish territory beyond the RWEPA is high wolf mortality. Within the RWEPA, the USFWS has worked closely with trappers and property owners to limit red wolf mortality (Wildlife Management Institute 2014). Outside the RWEPA, there are no restrictions on coyote hunting and sportsmen may be unaware that red wolves may be present in this landscape. Furthermore, a large percentage of wolves are killed due to gunshot in the RWEPA (USFWS 2007; Bartel and Rabon 2013). Dispersing wolves are often at greater vulnerability to human‐derived mortality, which can limit colonization (Haight et al. 1998; Boyd and Pletscher 1999; Murray et al. 2010). In south‐eastern Canada, the distribution of eastern wolves outside of protected areas is limited by high mortality (Benson et al. 2014).

### Interpretation of ancestry

Our inference of hybridization and the distribution of parental genotypes in this system depend on the use of genetic data. The fact that the taxonomic status and genetic history of the red wolf has been the subject of considerable debate has bearing on our conclusions. Some genetic data suggests the red wolf is not a monophyletic taxon and potentially the product of hybridization between coyotes and gray wolves (Wayne and Jenks 1991; Reich et al. 1999; vonHoldt et al. 2011). Other genetic research suggests it is similar to the eastern wolf (*C. lycaon* or *C. lupus lycaon*) found in south‐eastern Canada, and the two evolved from a North American lineage along with the coyote (Wilson et al. 2000; Chambers et al. 2012). Our research did not directly address this debate, but such controversy is relevant to our ability to assess this system. The potential hybrid ancestry of red wolves raises questions about the ability of our dataset to differentiate red wolves and identify hybrids using our dataset. However, it is clear based on our Bayesian analysis that there are two distinct genetic groups in this region, one of which corresponds to the red wolf population. This corroborates other studies using our suite of loci, similar sets of microsatellites, and genomewide that the modern red wolf population can be distinguished genetically from other canids (Wilson et al. 2000; Bohling and Waits 2011; vonHoldt et al. 2011; Bohling et al. 2013). While this may have little bearing on the assessment of the origin or history of the red wolf, in the narrow sense of assessing patterns of hybridization and identifying parental groups in North Carolina we feel we have sufficient resolution in our data.

Another issue is the use of arbitrary cut‐offs for the STRUCTURE *q*‐values for classifying hybrids. Although typical in many hybridization studies, setting hard thresholds can facilitate misinterpretation of admixed ancestry, especially in situations involving backcrossing (Vähä and Primmer 2006; Bohling et al. 2013). However, we believe our decision to use this cut‐off value had little impact on the interpretation of the results. The US Fish and Wildlife Service uses a cut‐off of 87.5% red wolf ancestry based on the pedigree of the wild population for classifying an individual as a red wolf (Stoskopf et al. 2005; Gese et al. 2015). It was based on the expected proportion of ancestry after two generations of backcrossing after an F1 event (i.e., 7/8 ancestry to red wolf pedigree). Using this cut‐off allows us to be consistent with their system. Only one individual in the empirical dataset had a *q*‐value between 0.9 and 0.65: one had a red wolf q‐value of 0.8644. On the other end of the spectrum, only four individuals had *q*‐values between 0.15 and 0.1 for the red wolf cluster. The observation that only a few individuals fell near our cut‐off values strengthens our confidence in the ability of the analysis to classify individuals based on ancestry coefficients. Also, we only used the cut‐off classification for the *χ* ^2^‐test of hybrid proportions across zones and Fig. 4. We relied solely on the *q*‐values themselves for the comparisons with the simulated datasets and the anova of red wolf ancestry across zones.

### Mechanisms of isolation

There are several potential explanations for the low proportion of hybrids we observed across the entire region. Selection against hybrids or low hybrid fitness is unlikely, for studies of both this red wolf system and other canid populations suggest that introgressed individuals are not hampered by outbreeding depression (Adams 2006; Kays et al. 2010; Monzón et al. 2014). Even if hybrids did have lower fitness, the presence of hybrid genotypes can be maintained by high levels of dispersal and intermixing (Barton and Hewitt 1985, 1989). Given that coyotes are found throughout the RWEPA, if the species were randomly mating we would expect a higher proportion of hybrids than we observed.

In mosaic hybrid zones, ecological segregation limits the potential for hybridizing species to interact (Howard 1986; Howard et al. 1993). For example, in south‐eastern Canada, the distribution of eastern wolves, coyotes, gray wolves, and their hybrids has been attributed to affinities for different habitat conditions (Sears et al. 2003; Benson et al. 2012, 2014), which may limit intermixing (Wilson et al. 2009; Rutledge et al. 2010a). However, the system in south‐eastern Canada, which is often compared to the red wolf system, exists in a region that contains larger blocks of protected and undisturbed habitat types than the fragmented landscape of eastern North Carolina. Red wolves within the RWEPA prefer open agricultural areas away from human development (Hinton and Chamberlain 2010; Dellinger et al. 2013), which coyotes also select for (Hinton et al. 2015b). Strong habitat selection may isolate red wolves to certain locations, but it seems unlikely to limit the distribution of coyotes given their adaptability. Plus, we found several coyotes within close proximity of red wolves (Fig. 4); hence, it is unlikely ecological segregation is limiting interactions.

We hypothesize that mate choice and assortative mating are playing a role in minimizing the extent of hybridization. Fewer hybrids were observed than predicted by Fredrickson and Hedrick (2006) under an optimistic scenario of assortative mating and interspecific aggression. USFWS biologists have suggested this based on field observations. There are differences in the body size (Hinton and Chamberlain 2014) and behavior (Phillips and Henry 1992; Hinton et al. 2013) between the two species that could facilitate positive assortative mating. Red wolves often display aggression toward sterile placeholder coyote and hybrids, displacing them from the landscape (Gese and Terletzky 2015). Additional research is needed to understand patterns of mate choice between canid species under natural conditions. If red wolves display assortative mating with respect to coyotes, it adds a novel perspective to the issue concerning the veracity of its designation as a species.

Any attempt to explain the role natural mechanisms play in regulating hybridization must also consider the role of human management in this system. A key question is how the interplay between natural and anthropogenic factors influences hybridization. Undoubtedly, aggressive removal and sterilization of coyotes and hybrids has limited the amount of genetic introgression into the red wolf population. However, it has not limited the ability of coyotes to colonize the RWEPA. Fecal DNA sampling detected many more coyotes than previously known by USFWS biologists, yet they did not reveal a comparable number of unknown hybrids. Despite heavy colonization by coyotes, hybridization is still infrequent, emphasizing the role of natural processes in limiting introgression. In addition, anthropogenic activities, such as gunshot mortality of breeding red wolves, can facilitate hybridization by altering social dynamics (Bohling and Waits 2015). This has been observed in other canid systems (Muñoz‐Fuentes et al. 2010; Rutledge et al. 2010b, 2012). Attributing the lack of hybrids, we observed solely to positive management neglects that these efforts are undermined by other anthropogenic forces.

### Implications for conservation

Our findings have implications for the future of red wolf conservation and other species threatened by hybridization. From the red wolf perspective, our results disprove the common perception that red wolves have been consumed by a genetic swarm and no longer exist as a distinct genetic entity in North Carolina (Wildlife Management Institute 2014, NC Wildlife Resources Commission 2015a,b). This is especially pertinent as the USFWS has been faced with calls to modify or even cancel the red wolf program due a perceived lack of success (Wildlife Management Institute 2014, NC Wildlife Resources Commission 2015a,b). Our results provide insights into the status of the red wolf population and hybridization dynamics that will inform these discussions.

One of the issues at the heart of the red wolf recovery effort is whether the red wolf is a ‘conservation‐reliant species’ that will forever require human intervention to persist in the wild (Scott et al. 2005, 2010; Goble et al. 2012). There is no feasible way to reduce the threat posed by hybridization with coyotes in North Carolina or anywhere in the red wolf historic range to zero. However, the red wolf adaptive management program in North Carolina has managed to establish a population and maintain the uniqueness of the wild red wolf gene pool despite two decades of interaction with coyotes (Gese et al. 2015). Management practices and policies were initially developed under the assumption that these species randomly interbreed when sympatric (Kelly et al. 1999; Stoskopf et al. 2005), yet this study and additional evidence suggest this is incorrect (Bohling and Waits 2015; Gese and Terletzky 2015; Gese et al. 2015; Hinton et al. 2015a). More importantly, although human management has undoubtedly helped keep the species in existence, it also counterbalances human actions that facilitate hybridization (Sparkman et al. 2011; Bohling and Waits 2015). Such complexity dictates a more nuanced perspective on ‘conservation‐reliant’ and development of recovery goals that acknowledge these factors (Redford et al. 2011; Rohlf et al. 2014). Hybridization may not be completely avoidable, but creating policies, partnerships, and strategies that allow red wolves to maintain their genomic uniqueness through a combination of natural and management‐assisted processes will be critical toward evaluating the viability of the species in the wild.

Although there have been attempts to manage the genetic composition of endangered populations in hybrid systems, none have been as comprehensive as the red wolf program. European nations are undertaking efforts to eradicate non‐native ruddy ducks (*Oxyura jamaicensis*) to protect white‐headed duck (*O. leucocephala*) populations from hybridization (Cranswick and Hall 2010). In the United States, there are initiatives to cull individuals from public and private bison (*Bison bison*) herds that possess cattle (*Bos taurus*) ancestry (Dratch and Gogan 2010). At most, these programs attempt to remove individuals with signatures of past introgression (e.g., bison, Siamese crocodiles [*Crocodylus siamensis*] [Fitzsimmons et al. 2002]) or eliminate the ‘undesirable’ hybridizing species (e.g., ruddy ducks in Europe). They do not, however, couple real‐time field monitoring with genetic analyses to limit introgression on a fine scale as has been practiced with the red wolf. Such management has been a ‘success’ in terms of fostering the existence of a unique red wolf genetic unit and limited numbers of hybrids. For other species in similar situations, the red wolf program can be used as a model to develop conservation strategies. Combining knowledge of natural processes, sound management practices, and innovative policies will be critical for guiding conservation biologists addressing hybridization as it concerns endangered species conservation.

## Data archiving statement

Data files containing microsatellite genotypes for individuals detected via noninvasive genetic sampling, geographic coordinates for fecal samples from which these individuals were identified, and coyote and red wolf genotypes used for simulating genotypes were submitted to the DRYAD Digital Repository (http://dx.doi.org/10.5061/dryad.g8j01).

## Supporting information


**Table S1.** Distribution of scat samples and genotyped individuals by sampling zone.
**Table S2.** Estimates for parameters of logistic functions fit to the distribution of *q*‐values generated by the empirical dataset and our four simulated scenarios.
**Figure S1.** Relationship between *F*_IS_ and *F*_ST_ for the 17 microsatellite loci utilized in this study.
**Figure S2.** Measures of (A) observed (*H*_O_) and unbiased expected heterozygosity (*H*_E_), (B) allelic richness, and (C) *F*_IS_ for each of the six sampling zones (note that Zones 3 and A have been combined).
**Figure S3.** Estimates of (A) mean Ln *P*(D) and (B) Δ*K* statistic produced by the STRUCTURE analysis for values of *K* from 1–10.
