# Supplementary material for: Describing a developing hybrid zone between red wolves and coyotes in eastern North Carolina, USA
Source: Evol Appl. 2016 Jun 1;9(6):791–804. doi: 10.1111/eva.12388 (PMC4908465; doi:10.1111/eva.12388)
Supplement: Supplementary file 1 — Table S1. Distribution of scat samples and genotyped individuals by sampling zone. Table S2. Estimates for parameters of logistic functions fit to the distribution of q‐values generated by the empirical dataset and our four simulated scenarios. Figure S1. Relationship between F IS and F ST for the 17 microsatellite loci utilized in this study. Figure S2. Measures of (A) observed (H O) and unbiased expected heterozygosity (H E), (B) allelic richness, and (C) F IS for each of the six sampling zones (note that Zones 3 and A have been combined). Figure S3. Estimates of (A) mean Ln P(D) and (B) ΔK statistic produced by the STRUCTURE analysis for values of K from 1–10. [file EVA-9-791-s001.docx]

**Supporting Information**

**Table S1**: Distribution of scat samples and genotyped individuals by sampling zone. Each row represents one of the six sampling zones. The columns indicate how many scats where collected from that zone, the number of those scats genotyped at six or more microsatellite loci, the number of genotypes that matched known canids, and unique genotypes. These values are further divided by the study for which they were collected. For scats, location is based on where the sample was collected. For individuals (both known and unique), location is based on the center of all locations where that individual was detected. Blank cells indicate that no sampling in those zones was performed; it is not equivalent to a value of zero.

|  | 2010 survey | | | | Dietary surveys | | | | 2008 survey | | | |
| --- | --- | --- | --- | --- | --- | --- | --- | --- | --- | --- | --- | --- |
| Zone | Scats | Genotyped | Known* | Unique^#^ | Scats | Genotyped | Known | Unique | Scats | Genotyped | Known | Unique |
| Zone 1 |  |  |  |  | 1551^@^ | 473 | 15 | 8 |  |  |  |  |
| Zone 2 |  |  |  |  |  |  | 32 | 42 |  |  |  |  |
| Zone 3/A | 230 | 76 | 4 | 35 |  |  | 16 | 32 |  |  |  |  |
| Zone B | 191 | 59 | 7 | 23 |  |  | 0 | 10 |  |  |  |  |
| Zone C | 79 | 21 | 0 | 17 |  |  |  |  | 814^%^ | 134 | 0 | 5 |
| Zone D |  |  |  |  |  |  |  |  |  |  | 0 | 76 |

*For 2010 the “Known” category includes both known canids tracked by field biologists and genotypes collected during the dietary surveys, for they were conducted prior to the 2010 field survey.

^#^Across all separate studies there were several unique genotypes (*n*=14) for which there was no recorded location data. These individuals were excluded from this table, for they were not used in any of the genetic analysis.

^@^GPS coordinates were only provided for those scats that were assigned to an individual. These surveys also covered Zones 1-B.

^%^The 2008 study area overlapped with a portion of the 2010 study area. These scats were not differentiated based on which sampling zone they were collected.

**Table S2**: Estimates for parameters of logistic functions fit to the distribution of *q*-values generated by the empirical dataset and our four simulated scenarios.

|  | Parameters | | |  |
| --- | --- | --- | --- | --- |
| Scenario | Asymptote (*a*) | Midpoint (*b*) | Slope (*c*) | Residual |
| Empirical | 0.999 | 232.917 | 0.364 | 0.023 |
| Random mating | 1.052 | 226.529 | 0.044 | 0.026 |
| Assortative mating | 1.024 | 227.121 | 0.065 | 0.022 |
| Red wolf challenges | 1.005 | 226.952 | 0.109 | 0.017 |
| Spatial mixing | 0.997 | 229.293 | 4.599 | 0.020 |

**Figure S1**: Relationship between F_IS_ and F_ST_ for the 17 microsatellite loci utilized in this study. Each locus is represented by an individual point and the solid line represents a linear regression with the corresponding linear model and correlation coefficient. Positive correlation between F_IS_ and F_ST_ is indicative of the Wahlund effect. Panel A presents the results from individuals sampled within the RWEPA. Panel B presents the results from individuals sampled from the areas outside the RWEPA.

**A.**


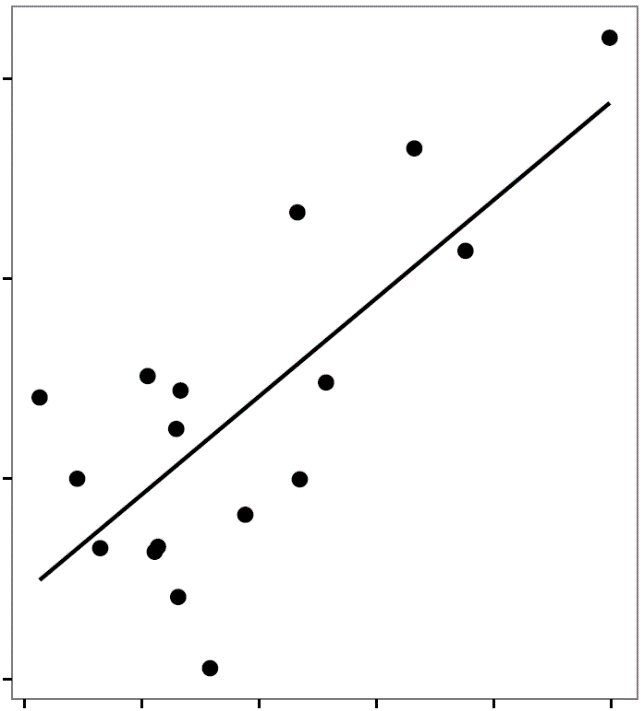


F_IS_=1.96*F_ST_+0.04, r=0.74

0.3

0.2

F_IS_

0.1

0.0

0.125

0.100

0.075

0.050

0.025

0.0

F_ST_

**B.**


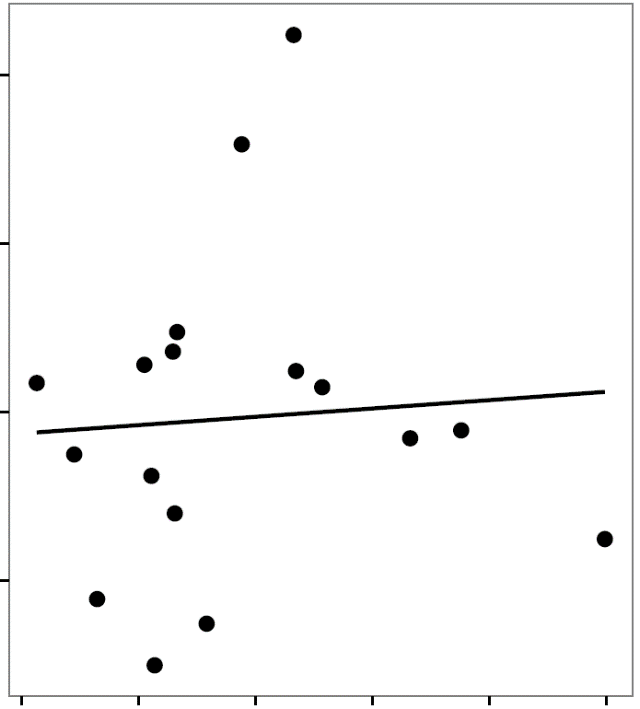


F_IS_=0.20*F_ST_+0.09, r=0.07

F_ST_

0.3

0.125

0.100

0.075

0.050

0.025

0.0

0.2

F_IS_

0.1

0.0

**Figure S2**: Measures of A.) observed (H_O_) and unbiased expected heterozygosity (H_E_), B.) allelic richness, and C.) F_IS_ for each of the six sampling zones (note that Zones 3 and A have been combined). In Panel A the light gray bars depict H_E_ and the darker bars depict H_O_. Panels for allelic richness and F_IS_ include 95% confidence intervals for each value. Note that sampling Zones 3 and A were combined for this analysis due to substantial spatial overlap.

0.8

0.6

0.4

0.2

0

A

H_O_

H_E_

Heterozygosity

B

8

6

Allelic richness

4

2

0

C

0.20

0.15

F_IS_

0.10

0.05

0

Zone 1

Zone 2

Zone 3

Zone B

Zone C

Zone D

**Figure S3**: Estimates of A.) mean Ln *P*(D) and B.) Δ*K* statistic produced by the STRUCTURE analysis for values of *K* from 1-10. The analysis involved five replicates of each *K*.

-13000

-14000

-15000

3000

2000

1000

0

1

2

3

4

5

6

7

8

9

10

*K*

Δ*K*

Mean Ln *P*(D)

A

B
